# Weak HIF-1alpha expression indicates poor prognosis in resectable pancreatic ductal adenocarcinoma

**DOI:** 10.1186/s12957-018-1432-4

**Published:** 2018-07-04

**Authors:** Joni Leppänen, Olli Helminen, Heikki Huhta, Joonas H. Kauppila, Joel Isohookana, Kirsi-Maria Haapasaari, Seppo Parkkila, Juha Saarnio, Petri P. Lehenkari, Tuomo J. Karttunen

**Affiliations:** 1 Cancer and Translational Medicine Research Unit, Medical Research Center Oulu, University of Oulu and Oulu University Hospital, 90014 Oulu, Finland; 2 Upper Gastrointestinal Surgery, Department of Molecular Medicine and Surgery, Karolinska Institutet, 17176 Stockholm, Sweden; 3 School of Medicine, University of Tampere, 33014 Tampere, Finland; 4 Fimlab Ltd, Tampere University Hospital, 33520 Tampere, Finland; 5 Department of Pathology, University of Oulu, PO-Box 5000, 90014 Oulu, Finland

**Keywords:** HIF-1alpha, Carbonic anhydrase 9, Pancreatic ductal adenocarcinoma

## Abstract

**Background:**

HIF-1alpha and CAIX proteins are commonly expressed under hypoxic conditions, but other regulatory factors have been described as well. Pancreatic ductal adenocarcinoma (PDAC) is characterized by hypoxia and strong stromal reaction and has a dismal prognosis with the currently available treatment modalities.

**Methods:**

We investigated the expression and prognostic role of HIF-1alpha and CAIX in PDAC series from Northern Finland (*n* = 69) using immunohistochemistry.

**Results:**

In our PDAC cases, 95 and 85% showed HIF-1alpha and CAIX expression, respectively. Low HIF-1alpha expression correlated with poor prognosis, and multivariate analysis identified weak HIF-1alpha intensity as an independent prognostic factor for PDAC-specific deaths (HR 2.176, 95% CI 1.216–3.893; *p* = 0.009). There was no correlation between HIF-1alpha and CAIX expression levels, and the latter did not relate with survival.

**Conclusions:**

Our findings are in contrast with previous research by finding an association between low HIF-1alpha and poor prognosis. The biological mechanisms remain speculative, but such an unexpected relation with prognosis and absence of correlation between HIF-1alpha and CAIX suggests that the prognostic association of HIF-1alpha may not directly be linked with hypoxia. Accordingly, the role of HIF-1alpha might be more complex than previously thought and the use of this marker as a hypoxia-related prognostic factor should be addressed with caution.

**Electronic supplementary material:**

The online version of this article (10.1186/s12957-018-1432-4) contains supplementary material, which is available to authorized users.

## Background

Pancreatic ductal adenocarcinoma (PDAC) is one of the deadliest cancers worldwide [1]. Surgical and oncological treatment of PDAC is arduous for the patient but still, the long-term survival rates are low [2]. Surgical resection [3], often in combination with adjuvant chemotherapy [4], is the only curative treatment of PDAC.

PDAC is characterized by hypoxia and neovascularization [5, 6]. Hypoxia-inducible factor 1 alpha (HIF-1alpha) is a protein expressed under hypoxic conditions [7]. HIF-1alpha regulates tumor angiogenesis [8], and high level of HIF-1alpha has been linked to tumor neoangiogenesis in PDAC [9–13]. Previous studies have indicated that strong HIF-1alpha expression associates with poor overall survival of patients with PDAC [9, 14–20]. Carbonic anhydrase 9 (CAIX) is a membrane-associated protein that is regulated by HIF-1alpha under hypoxic conditions [21]. Increased expression has been found in PDAC [22], and an association between abundance of CAIX and poor prognosis has been suggested [23].

The aim of the present study was to investigate the expression of and association between HIF-1alpha and CAIX expression and prognosis in a Finnish PDAC cohort.

## Methods

### Patients

Some 69 patients underwent surgery for PDAC in Oulu University Hospital during 1993–2011. For these patients, archival paraffinized specimens were collected from the pathology archive of the hospital. Clinical data was extracted from the patient records (Table 1). Statistics Finland provided complete survival data until the end of 2015. An expert gastrointestinal pathologist (TJK) confirmed the PDAC diagnosis. The majority of the patients underwent pancreaticoduodenectomy (i.e., Whipple procedure; *n* = 56) while two patients had distal pancreatectomy and 11 patients had total pancreatectomy. Macroscopic venous invasion was observed in six of the patients during the operation. Tumor grade was not determinable in 16 patients. Patients had a median age of 66 years at diagnosis, the range being 36–77 years. The median follow-up time in the cohort was 21 months (range 1–173 months). Of the 69 patients, 12 (17%) developed distant metastasis within 6 months of surgery, but stage at the time of surgery was used in the analyses. Samples of ischemic colon (*n* = 4) were used as a positive control for HIF-1alpha. The study was approved by the Oulu University Hospital Ethics Committee (EETTMK:81/2008) and by the National Authority for Medicolegal Affairs (VALVIRA).

**Table 1:** Baseline characteristics of 69 patients with resected pancreatic ductal adenocarcinoma

Patient clinical data	*n*/*N*	Percent
Age at diagnosis		
< 65	34/69	49
≥ 65	35/69	51
Sex		
Male	36/69	52
Female	33/69	48
Tumor size (mm)		
< 30	23/69	33
30–40	31/69	45.0
> 40	15/69	22
Tumor stage		
I	18/68	26
II	44/68	65
III–IV	6/68	9
Lymph nodes^*^		
Negative	34/68	50
Positive	34/68	50
HIF-1alpha		
Strong	28/64	44
Weak	36/64	56
BMI^**^		
< 25	19/46	41
> 25	27/46	59
Smoking		
Smoker^**^	14/33	42
Ex-smoker	7/33	21
Non-smoker	12/33	36
Alcohol usage^**^		
Heavy	7/24	29
Moderate-No	17/24	71
Chronic pancreatitis^***^		
Present	39/69	57
Absent	30/69	43

^*^Lymph node status and tumor stage were available from 68 patients

^**^BMI was missing from 22, smoking status from 36 patients, and alcohol usage from 45 patients

^***^Present or absent chronic pancreatitis reported pre-operatively in the patient records or in the histological analysis

### Immunohistochemistry

Immunohistochemistry was performed on representative tissue block sections, chosen using hematoxylin and eosin stainings. Commercial monoclonal mouse antibody against HIF-1alpha (NB100-105 IgG2b, Clone H1alpha67, Novus Biologicals, Littleton, CO) has previously been validated for use in formalin-fixed paraffin-embedded material [24–26] and was used at a dilution of 1:300. Dako Envision flex kit (Dako, Copenhagen, Denmark) with high-temperature antigen retrieval in Tris-EDTA buffer for 20 min (pH 9.0) was used for detection of antibody reaction and diaminobenzidine (Dako basic DAB-kit) as a chromogen. Dako Autostainer (Dako) was used for the stainings.

The previously described monoclonal antibody M75 was used to recognize the N-terminal domain of human CAIX [27], with normal rabbit serum acting as negative control. Immunohistochemical staining was performed using automated Lab Vision Autostainer 480 (ImmunoVision Technologies Co., Brisbane, CA) and polymer-based Power Vision+™ Poly-HRP IHC Kit reagents (ImmunoVision Technologies, Co.) in room temperature, as described earlier [28].

Three series of controls (primary antibody omitted, primary antibody replaced with mouse primary antibody isotype control, and staining with irrelevant CD3-antibody) were performed. Specimens of ischemic intestine were used as positive controls for HIF-1alpha staining.

### Histological analysis

Two (HIF-1alpha) or three investigators (CAIX), blinded to outcomes, evaluated the specimens as previously described [29, 30]. HIF-1alpha (*n* = 64) and CAIX (*n* = 65) stainings of PDAC could be evaluated due to availability of tissue section material. Separate assessment of adjacent normal pancreas was conducted, including normal duct epithelium and exocrine parenchyma (*n* = 35 and *n* = 39 for HIF-1alpha and CAIX, respectively). Separate assessment of membranous, cytoplasmic, and nuclear staining in the tumor cells was conducted with scoring of intensity on a four-point scales 0–3 (absent to strong). For statistical analysis, nuclear HIF-1alpha intensity and membranous CAIX intensity were divided by the median level into two groups, weak and strong. The percentage of positively stained cells was recorded using a five-point scale ranging from 0 to 4: 0 = < 1%, 1 = 1–10%, 2 = 11–49%, 3 = 50–80%, and 4 = > 80%. A score was calculated using a formula described earlier [26]: Score = [(1 + intensity)/3] × proportion of positive cells (0–4), resulting in score ranging from 0 to 5.33. This scoring allowed the percentage of stained cells to have greater impact on the score than staining intensity. The median score (2.167) was used to dichotomize the expression into two groups (weak and strong). The mean value of the separate assessments was used in the statistical analysis if the inter-observer difference < 1 step in intensity and < 30% in percentage. Greater discrepancies were jointly re-evaluated, resulting in a single score. According to these criteria, only one specimen needed re-evaluation for CAIX intensity.

### Statistical analysis

IBM SPSS statistics for Windows, version 22.0, Armonk, NY: IBM Corp, was used for all analyses. Nuclear HIF-1alpha and membranous CAIX intensity between cancerous tissue and adjacent normal pancreatic tissue was compared with Wilcoxon paired test. Differences between prognostic and clinicopathological variables (TNM staging, tumor grade, tumor size, BMI, and sex) were calculated with the chi-square test. Life tables were calculated with Kaplan-Meier method, and survival curves compared with log-rank test. Cox proportional hazards model provided hazard ratios (HRs) and 95% confidence intervals (CIs) for each variable. The following factors were analyzed: nuclear HIF-1alpha staining intensity (weak, or strong), age at diagnosis (< 65, or ≥ 65 years), sex (male or female) and tumor stage (I, II, or III–IV). Correlation between nuclear HIF-1alpha expression and membranous CAIX expression was obtained by Spearman’s non-parametrical correlation. *P* < 0.05 was considered statistically significant.

## Results

### HIF-1alpha and CAIX are expressed in normal pancreas and in pancreatic ductal adenocarcinoma

**Fig. 1:**
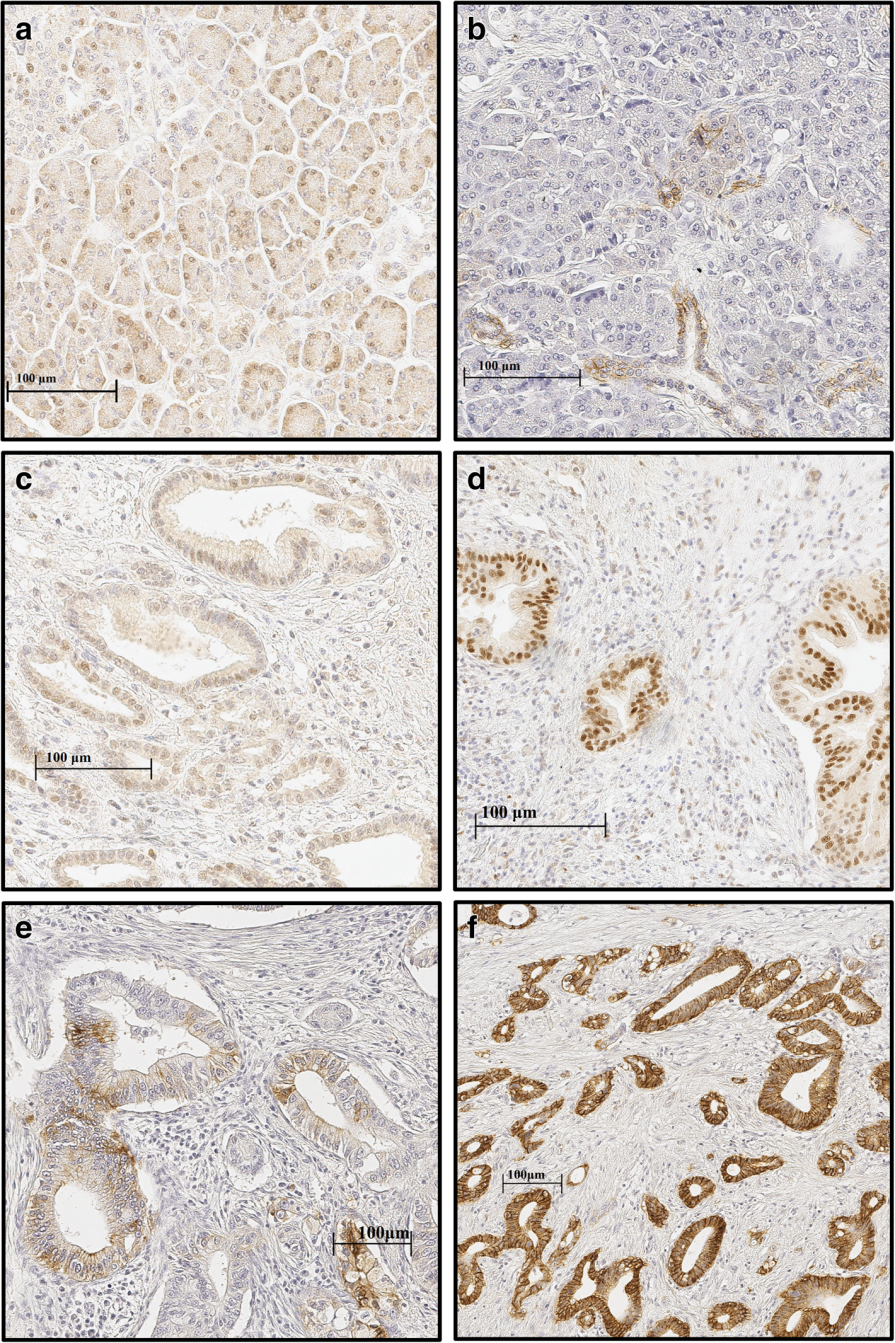
HIF-1alpha and CAIX expression in PDAC and adjacent normal pancreatic tissue. Adjacent normal exocrine pancreas with weak nuclear and cytoplasmic HIF-1alpha staining (**a**). Adjacent normal pancreatic tissue with weak membranous CAIX staining in the ducts and absent staining in exocrine pancreatic tissue (**b**). PDAC with weak (intensity 1) nuclear and cytoplasmic HIF-1alpha staining (**c**). PDAC with strong (intensity 3) nuclear and moderate (intensity 2) cytoplasmic HIF-1alpha staining (**d**). PDAC with weak to moderate (intensity 1–2) membranous CAIX staining (**e**). PDAC with strong (intensity 3) membranous CAIX staining (**f**)

For HIF-1alpha staining, normal pancreatic tissue adjacent to PDAC was present in 35/64 cases. In this adjacent normal pancreas, there was positive nuclear expression of HIF-1alpha in 19/35 (54%) cases and positive cytoplasmic HIF-1alpha expression in all of the studied cases (35/35). Nuclear and cytoplasmic staining was present in the exocrine cells and ductal epithelium. Positive nuclear HIF-1alpha staining was more prevalent and extensive in cancerous tissue, where it was found in 61/64 (95%) cases (mean proportion of positive cells 37%, SD 24, range 0–95, mean intensity 2.2, SD 0.7, range 0–3), as compared with the adjacent normal pancreatic tissue (mean proportion of positive cells 19%, SD 21, range 0–55, mean intensity 1.1, SD 1.1, range 0–3); (*p* < 0.001) **(**Fig. 1a, c, d).

In PDAC, cytoplasmic HIF-1alpha expression was present in 61/64 (95%) cases (mean proportion of positive cells 82%, SD 29, range 0–100, mean intensity 1.4, SD 0.6, range 0–3) and was more prevalent compared to adjacent normal pancreatic tissue (mean proportion of positive cells 95%, SD 17, range 0–100, mean intensity 1.9, SD 0.7, range 0–3); (*p* = 0.009) **(**Fig. 1a, c, d). Co-expression of nuclear and cytoplasmic staining was present in 60/64 cases. There was no correlation in HIF-1alpha expression between adjacent normal pancreas and carcinoma cells.

In CAIX stainings, adjacent normal pancreatic tissue was present in 39/65 cases. CAIX was expressed in normal pancreas in 27/39 (69%) cases (mean proportion 12.1%, SD 15.4, range 0–73, mean intensity 0.76, SD 0.56, range 0–2). The expression was mainly membranous and located in the ductal epithelial cells. In PDCA, CAIX was expressed in the cell membrane of the cancer cells (Fig. 1b, e, f), staining being present in 55/65 (85%) cases (mean proportion 38.8%, SD 29.9, range 0–100, mean intensity 1.65, SD 0.97, range 0–3). Membranous CAIX staining was significantly increased in cancerous tissue compared to adjacent normal pancreatic tissue (*p* < 0.001). There was no correlation in CAIX expression between adjacent normal pancreas and carcinoma cells.

### Control stainings for HIF-1alpha

In order to confirm performance of the HIF-1alpha staining and to exclude the possible bias caused by unspecific positive staining, samples of ischemic colon (*n* = 4) were stained with HIF-1alpha antibody for positive control. As expected, mucosal epithelium showed positive nuclear HIF-1alpha staining in the ischemic cells, with the staining intensity gradually increasing from absent to strong in the cells adjacent to ischemic necrosis (Additional file 1: Figure S1). For a negative control PDAC, samples showing high nuclear HIF-1alpha were stained with CD3 antibody (*n* = 3). CD3 staining was present only in the lymphocytes and not in the carcinoma cells (Additional file 1: Figure S1).

### Correlation between HIF-1alpha and CAIX

There was no correlation between nuclear or cytoplasmic HIF-1alpha expression intensity and membranous CAIX intensity in carcinoma cells. No correlation was found between HIF-1alpha and CAIX in adjacent normal pancreatic tissue. Also, no correlation in HIF-1alpha or CAIX between adjacent normal pancreas and carcinoma cells was observed.

### Clinicopathological variables and HIF-1alpha and CAIX expression

No correlation between nuclear HIF-1alpha expression and clinicopathological variables (TNM stage, tumor grade, tumor size, BMI, and sex) was found, Table 2. Males showed a weaker membranous CAIX staining compared to females (*p* = 0.038). There were no other correlations between membranous CAIX staining and the clinicopathological variables (Table 2).

**Table 2:** Intensity of HIF-1alpha and CAIX compared to clinicopathological variables in pancreatic ductal adenocarcinoma. Statistically significant *p* values are italicized

Variable	*n*/*N*	Nuclear HIF-1alpha intensity, *n*			*n*/*N*	Membranous CAIX intensity, *n*		
		Weak	Strong	*p*		Weak	Strong	*p*
pT								
T1	4/64	1	3	0.439	4/65	4	0	0.533
T2	22/64	11	11		22/65	15	7	
T3	32/64	20	12		33/65	23	10	
T4	6/64	4	2		6/65	5	1	
Lymph nodes								
Positive	31/64	16	15	0.556	32/65	23	9	0.823
Negative	32/64	19	13	*	32/65	23	9	*
Stage								
I	18/63	7	11	0.236	18/64	13	5	0.794
II	39/63	24	15	*	40/64	28	12	*
III–IV	6/63	4	2		6/64	5	1	
Tumor grade								
1	9/63	4	5	0.351	9/64	6	3	0.344
2	28/63	17	11	**	28/64	22	6	**
3	13/63	9	4		13/64	11	2	
Tumor size								
> 30 mm	42/64	23	19	0.475	43/65	30	13	0.370
< 30 mm	22/64	13	9		22/65	17	5	
BMI								
> 25	25/43	15	10	0.366	26/44	19	7	0.186
< 25	18/43	9	9	***	18/44	16	2	***
Sex								
Male	35/64	19	16	0.463	35/65	29	6	*0.038*
Female	29/64	17	12		30/65	18	12	

^*^Lymph node status was unavailable for one patient

^**^Tumor grade was missing from 16 patients

^***^BMI was available only for 47 patients

### BMI, smoking, alcohol consumption, or chronic pancreatitis and HIF-1alpha and CAIX expression

BMI, smoking, alcohol consumption, or chronic pancreatitis were not associated with HIF-1alpha or CAIX expression. Chronic pancreatitis did not affect patient survival (data not shown). Numbers and percentages of BMI, smoking status, alcohol usage, and chronic pancreatitis are summarized in Table 1.

### Survival, HIF-1alpha and CAIX expression: univariate and multivariate analysis

**Fig. 2:**
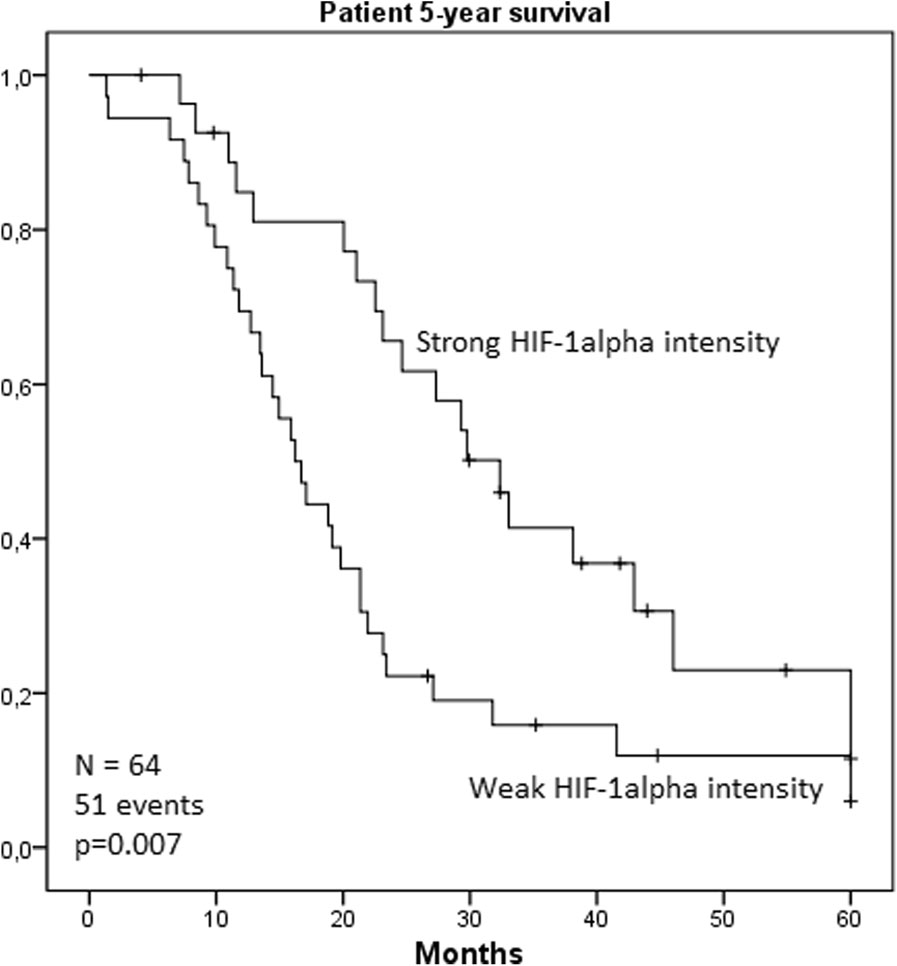
Kaplan-Meier curve visualizing the effect of weak nuclear HIF-1alpha intensity on patient 5-year survival. Mean survival time for patients with weak nuclear HIF-1alpha intensity (*n* = 36) was 21.5 months, as patients with strong nuclear HIF-1alpha intensity (*n* = 28) mean survival time was 34.2 months; (*p* = 0.007 log-rank)

The association between HIF-1alpha and CAIX expression and 5-year survival was analyzed. Mean 5-year survival was 12 months shorter in the group with weak nuclear HIF-1alpha intensity (21.5 months; 95% CI 15.9–27.0) as compared with strong intensity (34.2 months; 95% CI 27.0–41.3; *p* = 0.007, log-rank; Fig. 2). Multivariate analysis identified weak nuclear HIF-1alpha intensity as an independent prognostic factor for PDAC-specific deaths (HR 2.176, 95% CI 1.216–3.893, *p* = 0.009; Additional file 2: Table S1).

Similarly, using HIF-1alpha score to address the simultaneous impact of the intensity and extent of the staining, an association between weak HIF-1alpha expression and poor survival was found. Patients with a weak HIF-1alpha score had significantly shorter survival time (22.5 months; 95% CI 16.3–28.6) compared to patients with a strong score (31.5 months; 95% CI 24.7–38.2; *p* = 0.050). In multivariate analysis, weak HIF-1alpha score showed an increased point estimate for hazard ratio similar to HIF-1alpha intensity, but the association was not statistically significant (HR 1.7, 95%CI 0.985–3.031; Additional file 3: Table S2).

CAIX expression was not associated with 5-year survival (*p* = 0.393). Furthermore, cytoplasmic HIF-1alpha expression was not associated with 5-year survival (*p* = 0.930).

## Discussion

The results of the present study suggest that absent to weak nuclear HIF-1alpha expression might be an independent predictor of poor survival. No significant association between CAIX expression and survival or the expression levels of HIF-1 alpha and CAIX was found.

Limitations of the present study include its retrospective nature, relatively small sample size, and low number of T4 cases in the analysis due to the inoperable nature of these tumors **(**Table 2).

Several previous studies have proposed an association between strong expression of HIF-1alpha and poor prognosis in PDAC [14]. Similarly, increased levels of CAIX in PDAC [22, 31] and impact on poor prognosis have been suggested [11, 23]. Although association with strong expression of HIF-1alpha and adverse prognosis has been reported in some other cancer types as well [32–35], there are also contrary reports, including association of weak HIF-1alpha expression and poor prognosis in squamous cell carcinoma of the oral cavity [36–38] and breast cancer [39].

The mechanism linking high HIF-1alpha expression and good prognosis remains speculative. In previous studies, HIF-1alpha associated commonly with other hypoxia-related markers, such as CAIX, suggesting hypoxia-dependent HIF-1alpha expression [14]. The absence of correlation between HIF-1alpha and CAIX in PDAC cells in the present study supports the hypothesis that rather than being regulated by hypoxia, HIF-1alpha expression could be modified by other factors. These factors include alterations in various tumor suppressor genes and oncogenes [36, 40–45]. Previous studies have indicated that such oxygen-independent HIF-1alpha expression results in a diffuse expression pattern throughout the tumor. This staining pattern is typically not limited to ischemic areas. Such diffuse expression has been reported in brain tumors, breast cancer, and oropharyngeal cancers [46–49]. We hypothesize that the loss of HIF-1alpha in tumors with adverse prognosis could just be a marker of the simultaneous presence of multiple severe genetic aberrations in PDAC cells. Furthermore, abundant expression of HIF-1alpha and the associated good prognosis could be manifestations of fewer genetic and functional aberrations. In support of such interpretation, HIF-1alpha expression in the adjacent normal pancreatic tissue was common, 54% of the cases showing nuclear HIF-1alpha expression, and in all cases, HIF-1alpha was detected in cytoplasm. Furthermore, 69% of the cases showed positive CAIX expression in adjacent normal pancreatic tissue. We hypothesize that HIF-1alpha expression in the normal pancreatic cells indicates their physiological abilities to respond to the relative lack of oxygen. Furthermore, we suggest that HIF-1alpha expression in the cancer cells could indicate their physiological ability to respond to hypoxia. Accordingly, high HIF-1alpha expression in the cancer cells could be a marker of the less malignant nature of these cells. This matter remains hypothetical and further evidence to confirm the association between HIF-1alpha levels and mutations in the oncogenes and tumor suppressor genes is needed.

In previous studies, it has been shown that oxidative phosphorylation is associated with chemoresistance and aggressiveness in pancreatic cancer [50]. Cells use glucose in a process of glycolysis where ATP and lactate are formed. Another mechanism involves glycolysis, which is followed by pyruvate metabolism in the Krebs cycle and oxidative phosphorylation in the mitochondria [51]. Oxidative phosphorylation is reduced in many cancers, and association with poor outcome has been reported [52]. It has been shown that pancreatic cancer is metabolically heterogeneous. Furthermore, the number of tumor cells relying on oxidative phosphorylation is high in pancreatic cancer [53]. These tumor cells are typically highly metastatic and have more tumorigenic potential than the cells less reliant to oxidative phosphorylation [54]. Furthermore, studies investigating human colon carcinoma cells in vitro have shown that HIF-1alpha reduces oxidative phosphorylation [55]. The HIF-1alpha expression detected in our material could be an indicator for reduced oxidative phosphorylation and reduced tumorigenic properties. However, this hypothetical association needs to be assessed in further studies.

Methodological issues in previous studies could also contribute to the discrepant findings about the prognostic role of HIF-1alpha, as summarized in Table 3. Endogenous biotin present in normal and neoplastic pancreas can lead to false positive cytoplasmic or nuclear staining, thus possibly explaining many of the previous findings [56–58]. Furthermore, the use of negative control is not reported in all of the previous studies [14]. This, together with the application of different antibodies and considering cytoplasmic staining of HIF-1alpha in the survival analysis, could contribute to the previous discordant results **(**Table 3).

**Table 3:** Immunohistochemical detection methods in the original articles included in the meta-analysis by Ye et al. [14]

Paper	Patients (*n*)	Immunohistochemistry procedures	HIF-1alfa antibody source and clone	Biotin based	Prognostic effect of HIF-1alpha	Reference
Sun et al. 2007	58	Supervision™, negative controls	BA0912, Wuhan Boster, China	?	High nuclear and/or cytoplasmic HIF-1alpha ➔ poor overall survival	[10]
Zhu et al. 2013	63	Supervision™, negative controls	Not mentioned	?	High nuclear and/or cytoplasmic HIF-1alpha ➔ poor overall survival	[20]
Zhang et al. 2010	65	Biotinylated goat anti-mouse or anti-rabbit (Streptavidin/peroxidase), negative controls	Santa Cruz, CA, USA	Yes	High nuclear and/or cytoplasmic HIF-1alpha ➔ poor overall survival	[18]
Miyake et al. 2008	39	Avidin-biotin (Dako LSAB™), no negative controls mentioned	Novus Biologicals, Littleton, CO	Yes	High nuclear HIF-1alpha ➔ poor overall survival	[17]
Matsuo et al. 2014	100	Avidin-biotin, no negative controls mentioned	Not mentioned	Yes	High nuclear and/or cytoplasmic HIF-1alpha ➔ poor overall survival	[9]
Ide et al. 2007	41	Biotinylated anti-mouse, anti-rabbit antibody conjugated to a peroxidase-labeled dextran polymer (Dako EnVision+™), no negative control mentioned	Clone HI-67, Novus Biologicals, Littleton, CO	No	High nuclear and/or cytoplasmic HIF-1alpha ➔ worse disease free survival, no correlation between HIF-1alpha and overall survival	[16]
Spivak-Kroizman et al. 2013	129	Bond™ Polymer Refine Detection Kit, no negative control mentioned	BD Biosciences	No	High nuclear HIF-1alpha ➔ poor overall survival	[15]
Zhao et al. 2012	95	Streptavidin biotin-peroxidase, biotinylated secondary antibody, no negative control mentioned	sc-10790, Santa Cruz, CA, USA	Yes	HIF-1alpha localization not mentioned. High HIF-1alpha ➔ poor overall survival	[19]

## Conclusion

In summary, weak nuclear HIF-1alpha expression associated with poor survival of the patients with PDAC in our material. CAIX was overexpressed in PDAC, but did not correlate with HIF-1alpha expression levels or prognosis, suggesting that factors other than hypoxia could also contribute to regulation of HIF-1alpha levels in PDAC and explain the effect on survival.

## Additional files

Additional file 1: **Figure S1.** Positive and negative controls for HIF-1alpha staining. The HIF-1alpha staining pattern in ischemic colon sample is nuclear and shows an increase in intensity towards the surface in the mucosal epithelium (a.). PDAC sample with high nuclear HIF-1alpha intensity stained with CD3 antibody for negative control (b.). CD3 staining is located in the lymphocytes but not in the cancer cells. (TIF 5034 kb)

Additional file 2: **Table S1.** Multivariate analysis for the contribution of clinical factors of pancreatic ductal adenocarcinoma to mortality after controlling for other variables. Tested explanatory variables were nuclear HIF-1alpha staining intensity (weak and strong), age at the time of diagnosis (< 65 or ≥ 65 years), sex (male or female) and tumor stage (I, II or III-IV). (DOCX 14 kb)

Additional file 3: **Table S2.** Multivariate analysis for the contribution of clinical factors of pancreatic ductal adenocarcinoma to mortality after controlling for other variables. Tested explanatory variables were nuclear HIF-1alpha score (weak and strong), age at the time of diagnosis (< 65 or ≥ 65 years), sex (male or female) and tumor stage (I, II or III-IV). (DOCX 14 kb)

## Abbreviations

CAIX: Carbonic anhydrase
HIF-1alpha: Hypoxia-inducible factor 1 alpha
PDAC: Pancreatic ductal adenocarcinoma

## References

[1] Ryan DP, Hong TS, Bardeesy N (2014). Pancreatic adenocarcinoma. N Engl J Med.

[2] Gall TM, Tsakok M, Wasan H, Jiao LR (2015). Pancreatic cancer: current management and treatment strategies. Postgrad Med J.

[3] Distler M, Ruckert F, Hunger M, Kersting S, Pilarsky C, Saeger HD, Grutzmann R. Evaluation of survival in patients after pancreatic head resection for ductal adenocarcinoma. BMC Surg 2013; 13:12,2482–2413–12. 10.1186/1471-2482-13-12 PMC3639824 23607915

[4] Oettle H, Post S, Neuhaus P, Gellert K, Langrehr J, Ridwelski K, Schramm H, Fahlke J, Zuelke C, Burkart C, Gutberlet K, Kettner E, Schmalenberg H, Weigang-Koehler K, Bechstein WO, Niedergethmann M, Schmidt-Wolf I, Roll L, Doerken B, Riess H (2007). Adjuvant chemotherapy with gemcitabine vs observation in patients undergoing curative-intent resection of pancreatic cancer: a randomized controlled trial. JAMA.

[5] Duffy JP, Eibl G, Reber HA, Hines OJ (2003). Influence of hypoxia and neoangiogenesis on the growth of pancreatic cancer. Mol Cancer.

[6] Akakura N, Kobayashi M, Horiuchi I, Suzuki A, Wang J, Chen J, Niizeki H, Kawamura K, Hosokawa M, Asaka M (2001). Constitutive expression of hypoxia-inducible factor-1alpha renders pancreatic cancer cells resistant to apoptosis induced by hypoxia and nutrient deprivation. Cancer Res.

[7] Ratcliffe PJ, O'Rourke JF, Maxwell PH, Pugh CW (1998). Oxygen sensing, hypoxia-inducible factor-1 and the regulation of mammalian gene expression. J Exp Biol.

[8] Liao D, Johnson RS (2007). Hypoxia: a key regulator of angiogenesis in cancer. Cancer Metastasis Rev.

[9] Matsuo Y, Ding Q, Desaki R, Maemura K, Mataki Y, Shinchi H, Natsugoe S, Takao S (2014). Hypoxia inducible factor-1 alpha plays a pivotal role in hepatic metastasis of pancreatic cancer: an immunohistochemical study. J Hepatobiliary Pancreat Sci.

[10] Sun HC, Qiu ZJ, Liu J, Sun J, Jiang T, Huang KJ, Yao M, Huang C (2007). Expression of hypoxia-inducible factor-1 alpha and associated proteins in pancreatic ductal adenocarcinoma and their impact on prognosis. Int J Oncol.

[11] Couvelard A, O'Toole D, Leek R, Turley H, Sauvanet A, Degott C, Ruszniewski P, Belghiti J, Harris AL, Gatter K, Pezzella F (2005). Expression of hypoxia-inducible factors is correlated with the presence of a fibrotic focus and angiogenesis in pancreatic ductal adenocarcinomas. Histopathology.

[12] Shibaji T, Nagao M, Ikeda N, Kanehiro H, Hisanaga M, Ko S, Fukumoto A, Nakajima Y (2003). Prognostic significance of HIF-1 alpha overexpression in human pancreatic cancer. Anticancer Res.

[13] Kitada T, Seki S, Sakaguchi H, Sawada T, Hirakawa K, Wakasa K (2003). Clinicopathological significance of hypoxia-inducible factor-1alpha expression in human pancreatic carcinoma. Histopathology.

[14] Ye LY, Zhang Q, Bai XL, Pankaj P, Hu QD, Liang TB (2014). Hypoxia-inducible factor 1alpha expression and its clinical significance in pancreatic cancer: a meta-analysis. Pancreatology.

[15] Spivak-Kroizman TR, Hostetter G, Posner R, Aziz M, Hu C, Demeure MJ, Von Hoff D, Hingorani SR, Palculict TB, Izzo J, Kiriakova GM, Abdelmelek M, Bartholomeusz G, James BP, Powis G (2013). Hypoxia triggers hedgehog-mediated tumor-stromal interactions in pancreatic cancer. Cancer Res.

[16] Ide T, Kitajima Y, Miyoshi A, Ohtsuka T, Mitsuno M, Ohtaka K, Miyazaki K (2007). The hypoxic environment in tumor-stromal cells accelerates pancreatic cancer progression via the activation of paracrine hepatocyte growth factor/c-Met signaling. Ann Surg Oncol.

[17] Miyake K, Yoshizumi T, Imura S, Sugimoto K, Batmunkh E, Kanemura H, Morine Y, Shimada M (2008). Expression of hypoxia-inducible factor-1alpha, histone deacetylase 1, and metastasis-associated protein 1 in pancreatic carcinoma: correlation with poor prognosis with possible regulation. Pancreas.

[18] Zhang JJ, Wu HS, Wang L, Tian Y, Zhang JH, Wu HL (2010). Expression and significance of TLR4 and HIF-1alpha in pancreatic ductal adenocarcinoma. World J Gastroenterol.

[19] Zhao T, Gao S, Wang X, Liu J, Duan Y, Yuan Z, Sheng J, Li S, Wang F, Yu M, Ren H, Hao J (2012). Hypoxia-inducible factor-1alpha regulates chemotactic migration of pancreatic ductal adenocarcinoma cells through directly transactivating the CX3CR1 gene. PLoS One.

[20] Zhu GH, Huang C, Feng ZZ, Lv XH, Qiu ZJ (2013). Hypoxia-induced snail expression through transcriptional regulation by HIF-1alpha in pancreatic cancer cells. Dig Dis Sci.

[21] Wykoff CC, Beasley NJ, Watson PH, Turner KJ, Pastorek J, Sibtain A, Wilson GD, Turley H, Talks KL, Maxwell PH, Pugh CW, Ratcliffe PJ, Harris AL (2000). Hypoxia-inducible expression of tumor-associated carbonic anhydrases. Cancer Res.

[22] Kivela AJ, Parkkila S, Saarnio J, Karttunen TJ, Kivela J, Parkkila AK, Pastorekova S, Pastorek J, Waheed A, Sly WS, Rajaniemi H (2000). Expression of transmembrane carbonic anhydrase isoenzymes IX and XII in normal human pancreas and pancreatic tumours. Histochem Cell Biol.

[23] Li Y, Dong M, Sheng W, Huang L (2016). Roles of carbonic anhydrase IX in development of pancreatic cancer. Pathol Oncol Res.

[24] Ke S, Chen S, Dong Z, Hong CS, Zhang Q, Tang L, Yang P, Zhai J, Yan H, Shen F, Zhuang Z, Wen W, Wang H (2017). Erythrocytosis in hepatocellular carcinoma portends poor prognosis by respiratory dysfunction secondary to mitochondrial DNA mutations. Hepatology.

[25] Serrano-Oviedo L, Gimenez-Bachs JM, Nam-Cha SY, Cimas FJ, Garcia-Cano J, Sanchez-Prieto R, Salinas-Sanchez AS (2017). Implication of VHL, ERK5, and HIF-1alpha in clear cell renal cell carcinoma: molecular basis. Urol Oncol.

[26] Colbert LE, Fisher SB, Balci S, Saka B, Chen Z, Kim S, El-Rayes BF, Adsay NV, Maithel SK, Landry JC, Curran WJ (2015). High nuclear hypoxia-inducible factor 1 alpha expression is a predictor of distant recurrence in patients with resected pancreatic adenocarcinoma. Int J Radiat Oncol Biol Phys.

[27] Pastorekova S, Zavadova Z, Kostal M, Babusikova O, Zavada J (1992). A novel quasi-viral agent, MaTu, is a two-component system. Virology.

[28] Viikila P, Kivela AJ, Mustonen H, Koskensalo S, Waheed A, Sly WS, Pastorek J, Pastorekova S, Parkkila S, Haglund C (2016). Carbonic anhydrase enzymes II, VII, IX and XII in colorectal carcinomas. World J Gastroenterol..

[29] Huhta H, Helminen O, Kauppila JH, Takala H, Metsikko K, Lehenkari P, Saarnio J, Karttunen T (2015). Toll-like receptor 9 expression in the natural history of Barrett mucosa. Virchows Arch.

[30] Helminen O, Huhta H, Takala H, Lehenkari PP, Saarnio J, Kauppila JH, Karttunen TJ (2014). Increased Toll-like receptor 5 expression indicates esophageal columnar dysplasia. Virchows Arch.

[31] Juhasz M, Chen J, Lendeckel U, Kellner U, Kasper HU, Tulassay Z, Pastorekova S, Malfertheiner P, Ebert MP (2003). Expression of carbonic anhydrase IX in human pancreatic cancer. Aliment Pharmacol Ther.

[32] Yang SL, Ren QG, Wen L, Hu JL (2016). Clinicopathological and prognostic significance of hypoxia-inducible factor-1 alpha in lung cancer: a systematic review with meta-analysis. J Huazhong Univ Sci Technolog Med Sci.

[33] Huang M, Chen Q, Xiao J, Yao T, Bian L, Liu C, Lin Z (2014). Overexpression of hypoxia-inducible factor-1alpha is a predictor of poor prognosis in cervical cancer: a clinicopathologic study and a meta-analysis. Int J Gynecol Cancer.

[34] Lin S, Ma R, Zheng XY, Yu H, Liang X, Lin H, Cai XJ (2014). Meta-analysis of immunohistochemical expression of hypoxia inducible factor-1alpha as a prognostic role in gastric cancer. World J Gastroenterol..

[35] Ping W, Sun W, Zu Y, Chen W, Fu X (2014). Clinicopathological and prognostic significance of hypoxia-inducible factor-1alpha in esophageal squamous cell carcinoma: a meta-analysis. Tumour Biol.

[36] Fillies T, Werkmeister R, van Diest PJ, Brandt B, Joos U, Buerger H (2005). HIF-1alpha overexpression indicates a good prognosis in early stage squamous cell carcinomas of the oral floor. BMC Cancer.

[37] dos Santos M, Mercante AM, Louro ID, Goncalves AJ, de Carvalho MB, da Silva EH, da Silva AM (2012). HIF-1alpha expression predicts survival of patients with squamous cell carcinoma of the oral cavity. PLoS One.

[38] Beasley NJ, Leek R, Alam M, Turley H, Cox GJ, Gatter K, Millard P, Fuggle S, Harris AL (2002). Hypoxia-inducible factors HIF-1alpha and HIF-2alpha in head and neck cancer: relationship to tumor biology and treatment outcome in surgically resected patients. Cancer Res.

[39] Vleugel MM, Greijer AE, Shvarts A, van der Groep P, van Berkel M, Aarbodem Y, van Tinteren H, Harris AL, van Diest PJ, van der Wall E (2005). Differential prognostic impact of hypoxia induced and diffuse HIF-1alpha expression in invasive breast cancer. J Clin Pathol.

[40] Kamisawa T, Wood LD, Itoi T, Takaori K (2016). Pancreatic cancer. Lancet.

[41] Semenza GL (2003). Targeting HIF-1 for cancer therapy. Nat Rev Cancer.

[42] Laughner E, Taghavi P, Chiles K, Mahon PC, Semenza GL (2001). HER2 (neu) signaling increases the rate of hypoxia-inducible factor 1alpha (HIF-1alpha) synthesis: novel mechanism for HIF-1-mediated vascular endothelial growth factor expression. Mol Cell Biol.

[43] Zhong H, Chiles K, Feldser D, Laughner E, Hanrahan C, Georgescu MM, Simons JW, Semenza GL (2000). Modulation of hypoxia-inducible factor 1alpha expression by the epidermal growth factor/phosphatidylinositol 3-kinase/PTEN/AKT/FRAP pathway in human prostate cancer cells: implications for tumor angiogenesis and therapeutics. Cancer Res.

[44] Ravi R, Mookerjee B, Bhujwalla ZM, Sutter CH, Artemov D, Zeng Q, Dillehay LE, Madan A, Semenza GL, Bedi A (2000). Regulation of tumor angiogenesis by p53-induced degradation of hypoxia-inducible factor 1alpha. Genes Dev.

[45] Maxwell PH, Wiesener MS, Chang GW, Clifford SC, Vaux EC, Cockman ME, Wykoff CC, Pugh CW, Maher ER, Ratcliffe PJ (1999). The tumour suppressor protein VHL targets hypoxia-inducible factors for oxygen-dependent proteolysis. Nature.

[46] Zagzag D, Zhong H, Scalzitti JM, Laughner E, Simons JW, Semenza GL (2000). Expression of hypoxia-inducible factor 1alpha in brain tumors: association with angiogenesis, invasion. and progression Cancer.

[47] Aebersold DM, Burri P, Beer KT, Laissue J, Djonov V, Greiner RH, Semenza GL (2001). Expression of hypoxia-inducible factor-1alpha: a novel predictive and prognostic parameter in the radiotherapy of oropharyngeal cancer. Cancer Res.

[48] Bos R, van der Groep P, Greijer AE, Shvarts A, Meijer S, Pinedo HM, Semenza GL, van Diest PJ, van der Wall E (2003). Levels of hypoxia-inducible factor-1alpha independently predict prognosis in patients with lymph node negative breast carcinoma. Cancer.

[49] Kuijper A, van der Groep P, van der Wall E, van Diest PJ (2005). Expression of hypoxia-inducible factor 1 alpha and its downstream targets in fibroepithelial tumors of the breast. Breast Cancer Res.

[50] Ashton TM, McKenna WG, Kunz-Schughart LA, Higgins GS (2018). Oxidative phosphorylation as an emerging target in cancer therapy. Clin Cancer Res.

[51] Vander Heiden MG, DeBerardinis RJ (2017). Understanding the intersections between metabolism and cancer biology. Cell.

[52] Yu M (2011). Generation, function and diagnostic value of mitochondrial DNA copy number alterations in human cancers. Life Sci.

[53] Viale A, Pettazzoni P, Lyssiotis CA, Ying H, Sanchez N, Marchesini M, Carugo A, Green T, Seth S, Giuliani V, Kost-Alimova M, Muller F, Colla S, Nezi L, Genovese G, Deem AK, Kapoor A, Yao W, Brunetto E, Kang Y, Yuan M, Asara JM, Wang YA, Heffernan TP, Kimmelman AC, Wang H, Fleming JB, Cantley LC, DePinho RA, Draetta GF (2014). Oncogene ablation-resistant pancreatic cancer cells depend on mitochondrial function. Nature.

[54] Viale A, Corti D, Draetta GF (2015). Tumors and mitochondrial respiration: a neglected connection. Cancer Res.

[55] Papandreou I, Cairns RA, Fontana L, Lim AL, Denko NC (2006). HIF-1 mediates adaptation to hypoxia by actively downregulating mitochondrial oxygen consumption. Cell Metab.

[56] Bussolati G, Gugliotta P, Volante M, Pace M, Papotti M (1997). Retrieved endogenous biotin: a novel marker and a potential pitfall in diagnostic immunohistochemistry. Histopathology.

[57] Duhamel RC, Johnson DA (1985). Use of nonfat dry milk to block nonspecific nuclear and membrane staining by avidin conjugates. J Histochem Cytochem.

[58] Vosse BA, Seelentag W, Bachmann A, Bosman FT, Yan P (2007). Background staining of visualization systems in immunohistochemistry: comparison of the Avidin-Biotin Complex system and the EnVision+ system. Appl Immunohistochem Mol Morphol.

