# Supplementary figures and images for: Weak HIF-1alpha expression indicates poor prognosis in resectable pancreatic ductal adenocarcinoma
Source: World J Surg Oncol. 2018 Jul 4;16:127. doi: 10.1186/s12957-018-1432-4 (PMC6033289; doi:10.1186/s12957-018-1432-4)

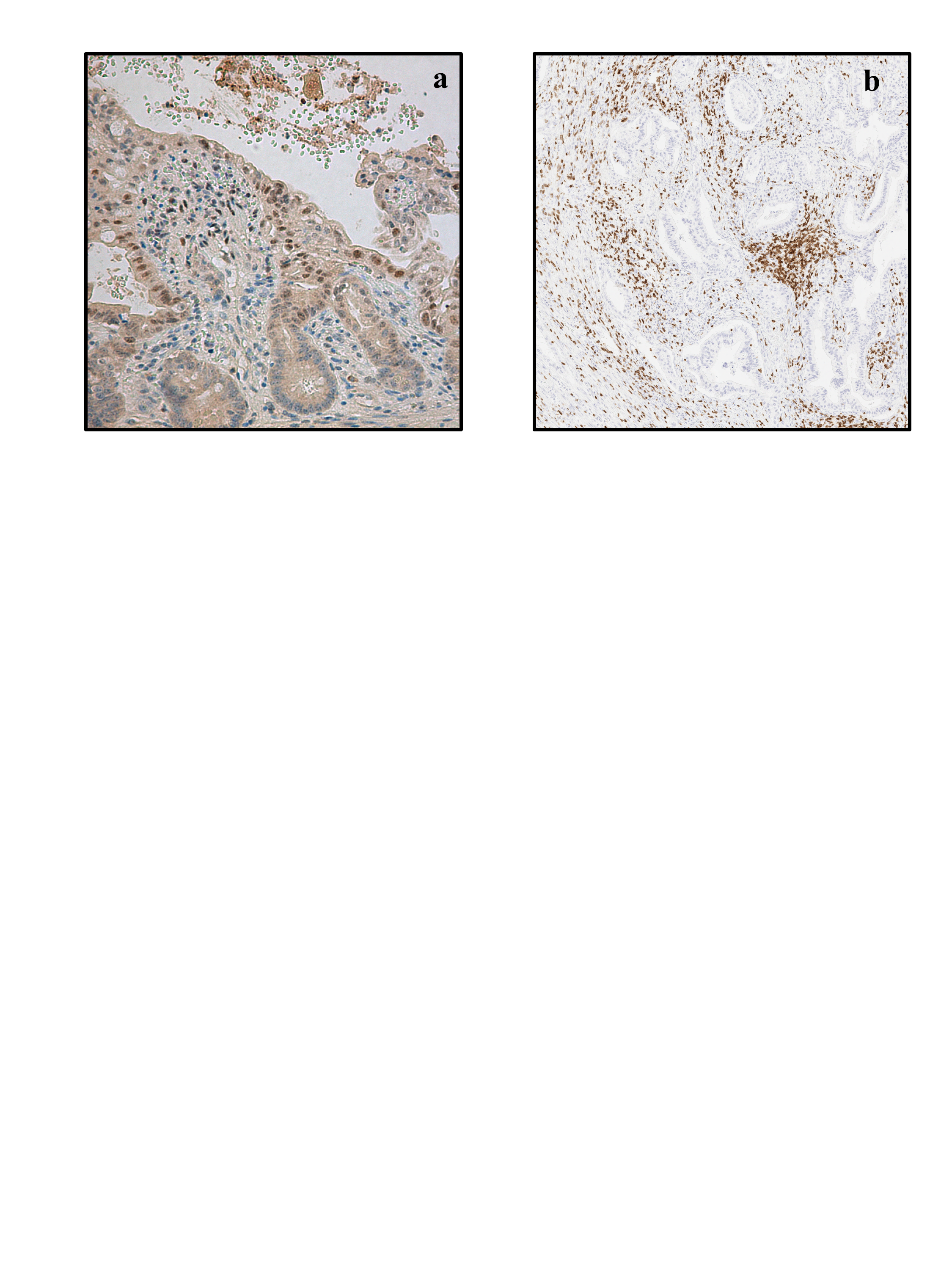

Supplement: Supplementary file 1 — Figure S1. Positive and negative controls for HIF-1alpha staining. The HIF-1alpha staining pattern in ischemic colon sample is nuclear and shows an increase in intensity towards the surface in the mucosal epithelium (a.). PDAC sample with high nuclear HIF-1alpha intensity stained with CD3 antibody for negative control (b.). CD3 staining is located in the lymphocytes but not in the cancer cells. (TIF 5034 kb) [file 12957_2018_1432_MOESM1_ESM.tif]
