# Supplementary material for: Weak HIF-1alpha expression indicates poor prognosis in resectable pancreatic ductal adenocarcinoma
Source: World J Surg Oncol. 2018 Jul 4;16:127. doi: 10.1186/s12957-018-1432-4 (PMC6033289; doi:10.1186/s12957-018-1432-4)
Supplement: Supplementary file 2 — Table S1. Multivariate analysis for the contribution of clinical factors of pancreatic ductal adenocarcinoma to mortality after controlling for other variables. Tested explanatory variables were nuclear HIF-1alpha staining intensity (weak and strong), age at the time of diagnosis (< 65 or ≥ 65 years), sex (male or female) and tumor stage (I, II or III-IV). (DOCX 14 kb) [file 12957_2018_1432_MOESM2_ESM.docx]

**Table S1** Multivariate analysis for the contribution of clinical factors of pancreatic ductal adenocarcinoma to mortality after controlling for other variables. Tested explanatory variables were nuclear HIF-1alpha staining intensity (weak and strong), age at the time of diagnosis (<65 or ≥65 years), sex (male or female) and tumor stage (I, II or III-IV).

| Variables | p | HR | 95% CI |
| --- | --- | --- | --- |
| HIF-1alpha |  |  |  |
| Strong |  | 1 |  |
| Weak | **0.009** | **2.176** | **1.216 – 3.893** |
|  |  |  |  |
| Age |  |  |  |
| ≥65 |  | 1 |  |
| <65 | 0.914 | 0.970 | 0.554 – 1.698 |
|  |  |  |  |
| Sex |  |  |  |
| Female |  | 1 |  |
| Male | 0.267 | 1.384 | 0.780 – 2.456 |
|  |  |  |  |
| Stage |  |  |  |
| I |  | 1 |  |
| II | 0.656 | 0.801 | 0.301 – 2.129 |
| III-IV | 0.647 | 0.812 | 0.334 – 1.977 |
