# Supplementary material for: Weak HIF-1alpha expression indicates poor prognosis in resectable pancreatic ductal adenocarcinoma
Source: World J Surg Oncol. 2018 Jul 4;16:127. doi: 10.1186/s12957-018-1432-4 (PMC6033289; doi:10.1186/s12957-018-1432-4)
Supplement: Supplementary file 3 — Table S2. Multivariate analysis for the contribution of clinical factors of pancreatic ductal adenocarcinoma to mortality after controlling for other variables. Tested explanatory variables were nuclear HIF-1alpha score (weak and strong), age at the time of diagnosis (< 65 or ≥ 65 years), sex (male or female) and tumor stage (I, II or III-IV). (DOCX 14 kb) [file 12957_2018_1432_MOESM3_ESM.docx]

**Table S2** Multivariate analysis for the contribution of clinical factors of pancreatic ductal adenocarcinoma to mortality after controlling for other variables. Tested explanatory variables were nuclear HIF-1alpha score (weak and strong), age at the time of diagnosis (<65 or ≥65 years), sex (male or female) and tumor stage (I, II or III-IV).

| Variables | p | HR | 95% CI |
| --- | --- | --- | --- |
| HIF-1alpha score |  |  |  |
| Strong |  | 1 |  |
| Weak | 0.057 | 1.728 | 0.985 – 3.031 |
|  |  |  |  |
| Age |  |  |  |
| ≥65 |  | 1 |  |
| <65 | 0.914 | 0.979 | 0.559 – 1.714 |
|  |  |  |  |
| Sex |  |  |  |
| Female |  | 1 |  |
| Male | 0.303 | 1.351 | 0.762 – 2.396 |
|  |  |  |  |
| Stage |  |  |  |
| I |  | 1 |  |
| II | 0.592 | 0.766 | 0.289 – 2.030 |
| III-IV | 0.732 | 0.856 | 0.353 – 2.078 |
